# Integrated analysis of long non-coding RNAs and mRNAs associated with glaucoma *in vitro*

**DOI:** 10.3389/fendo.2023.1087442

**Published:** 2023-02-27

**Authors:** Mengling You, Rong Rong, Zhou Zeng, Cong Fan, Haibo Li, Qian Yang, Dan Ji

**Affiliations:** ^1^ Department of Ophthalmology, Xiangya Hospital, Central South University, Changsha, China; ^2^ Hunan Key Laboratory of Ophthalmology, Xiangya Hospital, Central South University, Changsha, China; ^3^ National Clinical Research Center for Geriatric Disorders, Xiangya Hospital, Central South University, Changsha, China

**Keywords:** long non-coding RNA, mRNA, glaucoma, co-expression network, competitive endogenous RNA

## Abstract

**Introduction:**

In recent years, the biological functions and important roles of long non-coding RNAs (lncRNAs) have been widely reported in many diseases. Although glaucoma is the leading cause of blindness worldwide, the specific mechanisms of lncRNAs in the pathogenesis and progression of glaucoma remain unclear. Our research aims to elucidate the differentially expressed lncRNAs and mRNAs in glaucoma and to provide a basis for further exploration of the specific mechanism of action of lncRNAs in the progression of glaucoma.

**Methods:**

We performed RNA sequencing on samples from a pressurized model of R28 cells and performed bioinformatics analyses on the sequencing results. The expression consistency of lncRNAs in clinical samples from patients with glaucoma or cataracts was detected using real-time quantitative polymerase chain reaction (RT-qPCR).

**Results:**

RNA sequencing results showed that lncRNAs in cluster 5 were upregulated with increasing stress after typing all significantly altered lncRNAs using k-means in a cellular stress model. KEGG analysis indicated that they were associated with neurodegenerative diseases. Differentially expressed lncRNAs were verified by RT-qPCR, and the lncRNA expression levels of AC120246.2 and XLOC_006247 were significantly higher in the aqueous humor (AH) of patients with glaucoma than in those with cataracts. For LOC102551819, there was almost no expression in the AH and trabecular meshwork in patients with glaucoma but high expression was observed in the iris.

**Conclusion:**

Our research proposes potential diagnostic or intervention targets for clinical applications as well as a theoretical basis for more in-depth research on the function of lncRNAs in glaucoma.

## 1 Introduction

Glaucoma is a heterogeneous group of optic neuropathies characterized by the progressive loss of retinal ganglion cells (RGCs), thinning of the retinal nerve fiber layer, and vision loss (1). The pathogenesis of glaucoma is complex and diverse, involving pathologically high intraocular pressure (ph-IOP), microcirculation disorders, glutamate excitotoxicity, and immune abnormalities, which contribute to the primary causes of blindness worldwide (2–4). Currently, it is predicted that there will be 111.8 million patients with glaucoma aged 40–80 years worldwide by 2040 (5). Moreover, the early symptoms of glaucoma are mild and are often only found in the middle and late stages of the disease, which leads to delayed diagnosis. This puts significant pressure on social economy and public health. However, the pathogenic mechanisms underlying glaucoma are still not fully understood.

The continuous innovation in sequencing technology and development of genetic engineering has led to non-coding RNAs (ncRNAs) gradually receiving widespread attention. It is estimated that ncRNAs account for approximately 60% of the genetic material in the human genome (6). Additionally, more studies have revealed the diverse biological effects of ncRNAs in human developmental processes and diseases. Long non-coding RNAs (lncRNAs) are members of the ncRNA family and are characterized by a length greater than 200 nucleotides. The secondary structure of lncRNAs allows them to bind to certain proteins to facilitate chromatin remodeling and modification as well as the linear control of transcription factors. To date, various lncRNAs have been found to be differentially expressed in the aqueous humor, trabecular meshwork, iris and retinal cells, and venous blood of patients with glaucoma (7–9). However, the study of ncRNAs in glaucoma is still incomplete, and although their reported mechanism is mainly limited to competing endogenous RNA, it is generally thought that lncRNAs can bind related proteins or directly regulate mRNAs and encode short peptides to exert biological roles (10). Therefore, more in-depth exploration is required to understand how lncRNAs participate in both normal and pathogenic mechanisms.

We performed RNA sequencing on samples from a pressurized model of R28 cells (a retinal precursor cell line) that simulated the pathological process of acute high pressure in glaucoma. We also conducted a correlation analysis on the differentially expressed biological processes and signaling pathways to predict the network interaction of related lncRNA-miRNA-mRNA. We subsequently identified lncRNAs that showed significant changes in expression in clinical samples from glaucoma patients. In conclusion, our results provide novel targets for the clinical diagnosis and treatment of glaucoma and valuable information to support further in-depth studies of lncRNAs in glaucoma research.

## 2 Methods

### 2.1 R28 cell line cultivation

The R28 retinal cell line, an adherent retinal precursor cell line derived from the rat retina and is widely used in *in vitro* studies, was used in this study. It was provided by the Department of Anatomy and Neurobiology of Central South University (Changsha, China). R28 cells were cultured in Dulbecco’s modified Eagle’s medium (DMEM, Thermo Fisher Scientific, USA) with 10% fetal bovine serum (FBS, Thermo Fisher Scientific, USA), and 1% penicillin and streptomycin (NEST Biotechnology, Wuxi, China) at 37°C with an atmosphere containing 5% CO_2_. Before model construction, R28 cells were assessed by mycoplasma detection (MD001, Yisemed, Shanghai, China).

### 2.2 Glaucoma cellular model construction

To prepare the polyacrylamide hydrogel, we followed two published protocols (11, 12). Young’s moduli of the gels were measured using AFM. These moduli were 1.1, 2.5, 11.9, 34.4, and 50 kPa at bisacrylamide concentrations of 0.04%, 0.1%, 0.5%, 1.3%, and 2.08%, respectively. Rat R28 cells were seeded to confluency onto the gels.

### 2.3 Clinical sample collection

AH was obtained from patients with age-related cataract and primary angle-closure glaucoma (PACG). The iris and trabecular meshwork samples were collected from patients with POAG and PACG. All patients were operated on by the same experienced doctor in Xiangya Hospital (Changsha, China). Importantly, these patients did not have ocular surface disease, other optic nerve diseases, inflammatory diseases, or systemic diseases. The decision is taken by at least two clinicians with regard to the diagnosis of cataract and glaucoma (**Table S1**). This clinical portion of the study was approved by the Ethics Committee of Xiangya Hospital of Central South University, and written informed consent was obtained from all participating patients.

### 2.4 RNA-seq

Total RNA was extracted using the hot phenol method. The RNA was further purified with two phenol‐chloroform treatments and then treated with RQ1 DNase (Promega, Madison, WI) to remove possible DNA contamination. The quality and quantity of the purified RNA were determined by measuring the absorbance at 260 nm/280 nm (A260/A280) using SmartSpec Plus (BioRad). The RNA integrity was further verified with 1.5% agarose gel electrophoresis.

For each sample, 2 μg total RNA were used for RNA-seq library preparation. Polyadenylated RNAs were purified and concentrated with oligo (dT) – conjugated magnetic beads (Invitrogen, Carlsbad, CA) before directional library preparation. The purified RNAs were then iron fragmented at 95°C followed by end repair and five adapter ligation. Reverse transcription was performed with an RT primer containing a three adapter sequences and a randomized hexamer. Complementary DNAs (cDNA) were purified, amplified, and stored at −80°C until sequencing.

### 2.5 Differentially expressed genes and lncRNA analysis

The raw paired-end reads were trimmed and qualitycontrolled with SeqPrep (https://github.com/jstjohn/SeqPrep) and Sickle (https://github.com/najoshi/sickle). The clean reads were aligned to a Rat genome DatabaseV6 using HISAT2 (V2.1.0) and using bowtie2 (V2.2.9). The mapped reads of each sample were assembled by StringTie (V1.3.3b) in a reference-based approach. Finally, assembled transcripts were annotated by Cuffcompare program from the Cufflflinks (V2.2.1). The R Bioconductor package edgeR (13) was used to select differentially expressed genes (DEGs). A false discovery rate < 0.05 and fold change >2 or < 0.5 were set as the cut-off criteria for identifying DEGs and lncRNAs.

### 2.6 GO and KEGG analyses

To identify functional categories of DEGs, gene ontology (GO) analysis was performed using KOBAS3.0 software (http://kobas.cbi.pku.edu.cn), and the Kyoto Encyclopedia of Genes and Genomes (KEGG) database was used for pathway analysis to identify the significant enrichment of different molecular pathways using KOBAS3.0 software (http://www.genome.jp/kegg). The hypergeometric test and Benjamini-Hochberg FDR controlling procedure were used to define the enrichment of each term.

### 2.7 Co-expression network construction

Based on the expression of each mRNA and DElncRNA, the Pearson’s correlation coefficient (PCC) and P-value were obtained for each mRNA-DElncRNA pair. Then we filtered the result using a given threshold, with an absolute correlation coefficient of no less than 0.6 and P-value < 0.05. In addition to the positive correlation pairs, negative pairs with correlation coefficients less than 0 were also included. The filtered gene pairs were used to create the expression network. For each differentially expressed DElncRNA, we obtained the expressed genes from upstream and downstream regions within 10,000 bases. The genes were overlapped with co-expressed genes to obtain lncRNA targets. The co-expression network was illustrated using Cytoscape software (available online: https://cytoscape.org).

### 2.8 Competing endogenous RNA network construction

The lncRNAs and mRNAs were selected to predict miRNA targets using miRbase. Then, the miRNAs obtained from the predictions were screened with the miRanda and TargetScan programs. Afterwards, lncRNAs and mRNAs with miRNA recognition elements (MREs) for targeted miRNAs were predicted using RNA22. The competitive endogenous RNA (ceRNA) network was established and illustrated using Cytoscape software (14).

### 2.9 Quantitative real-time polymerase chain reaction (qRT–PCR)

Total RNA was extracted from the control and glaucoma patients by using TRIzol^®^ Reagent (Invitrogen, Carlsbad, CA, United States). First-strand cDNA for quantitative real-time PCR (qRT-PCR) analysis was obtained from 1 μg total RNA using an oligo primer and the UEIris II RT-PCR System and a First-Strand cDNA Synthesis Kit (US Everbright Inc, R2028, Suzhou, China) according to the manufacturer’s instructions. Real-time PCR was performed with a 2 × SYBR Green qPCR Master Mix (US Everbright Inc, S2014, Suzhou, China) using a 7500 FAST real-time PCR system (Applied Biosystems, Foster City, CA, United States). The expression of lncRNAs was calculated by the 2−ΔΔCt method. The forward and reverse primers for lncRNAs are shown in **Supplementary Table S2**.

### 2.10 Statistical analysis

Cluster3.0 and JavaTreeView were used to draw heat maps of gene and sample clusters. k-means were also used to cluster differently expressed model genes. Data are presented as mean ± standard deviation. All experiments were performed in triplicate. The statistical significance of differences between groups was calculated with the Student’s t-test in GraphPad Prism 7 software (GraphPad Software, La Jolla, CA, USA). All statistical tests were two-tailed, and a P-value < 0.05 was considered statistically significant. P-values < 0.05, < 0.01, and < 0.001 are indicated by *, **, and ***, respectively.

## 3 Results

### 3.1 mRNA and lncRNA differential expression in glaucoma

**Figure 1:**
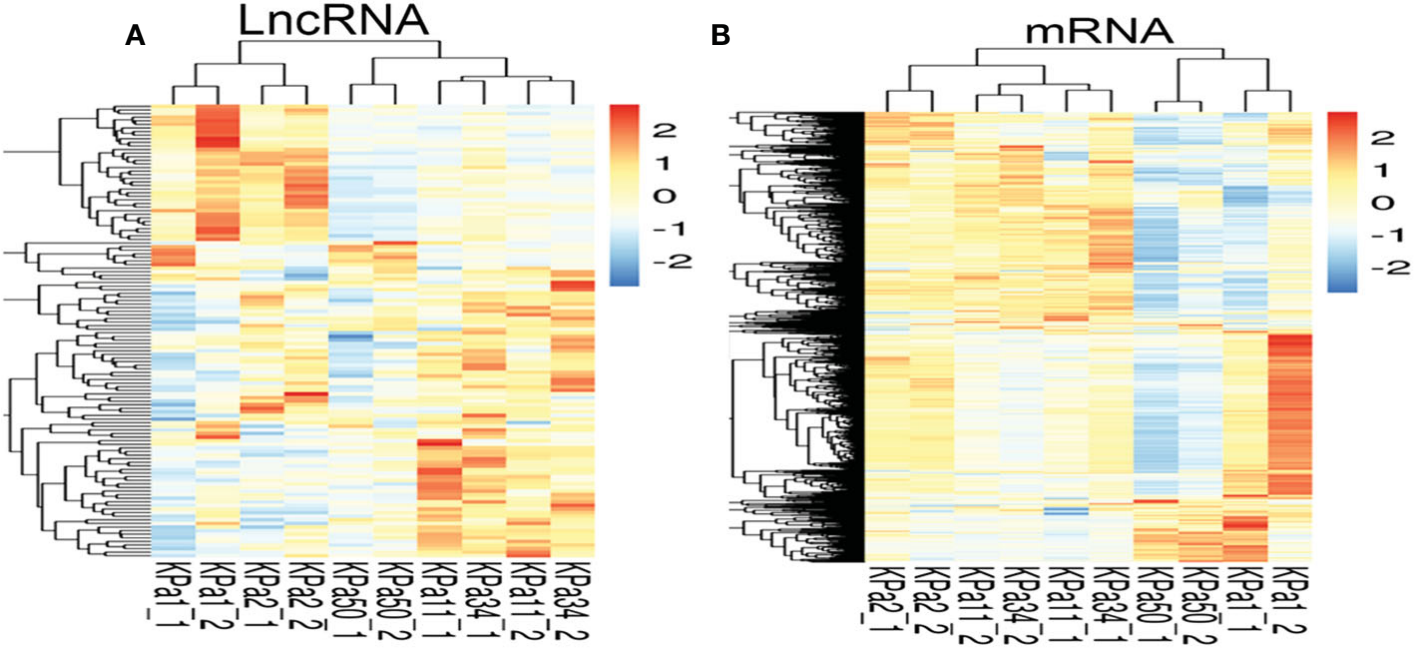
**(A, B)** Heat maps of mRNA and lncRNA(compare with 1 kpa). Each row represents one mRNA **(A)** or lncRNA **(B)**, and each column represents one different pressure value for the cell samples. Relative expression of mRNA or lncRNA is represented on a color scale. Red color indicates upregulation and blue indicates downregulation; 2, 0, -2, and 1.5, 0, -1.5 indicate fold changes in the corresponding spectrum.

lncRNAs and mRNAs possibly involved in the occurrence and development of glaucoma were identified by analyzing the lncRNA and mRNA expressed in the cellular stress model using RNA-seq. The expression of lncRNAs was divided into two types in the cell model. One type of lncRNA showed a gradual decrease in expression as the pressure increased, while the other type of lncRNA increased initially and then gradually decreased as the pressure increased. However, the overall trend observed was an increase in lncRNA and mRNA expression (**Figures 1A, B**).

### 3.2 GO and KEGG analysis of differentially expressed genes in glaucoma models

**Figure 2:**
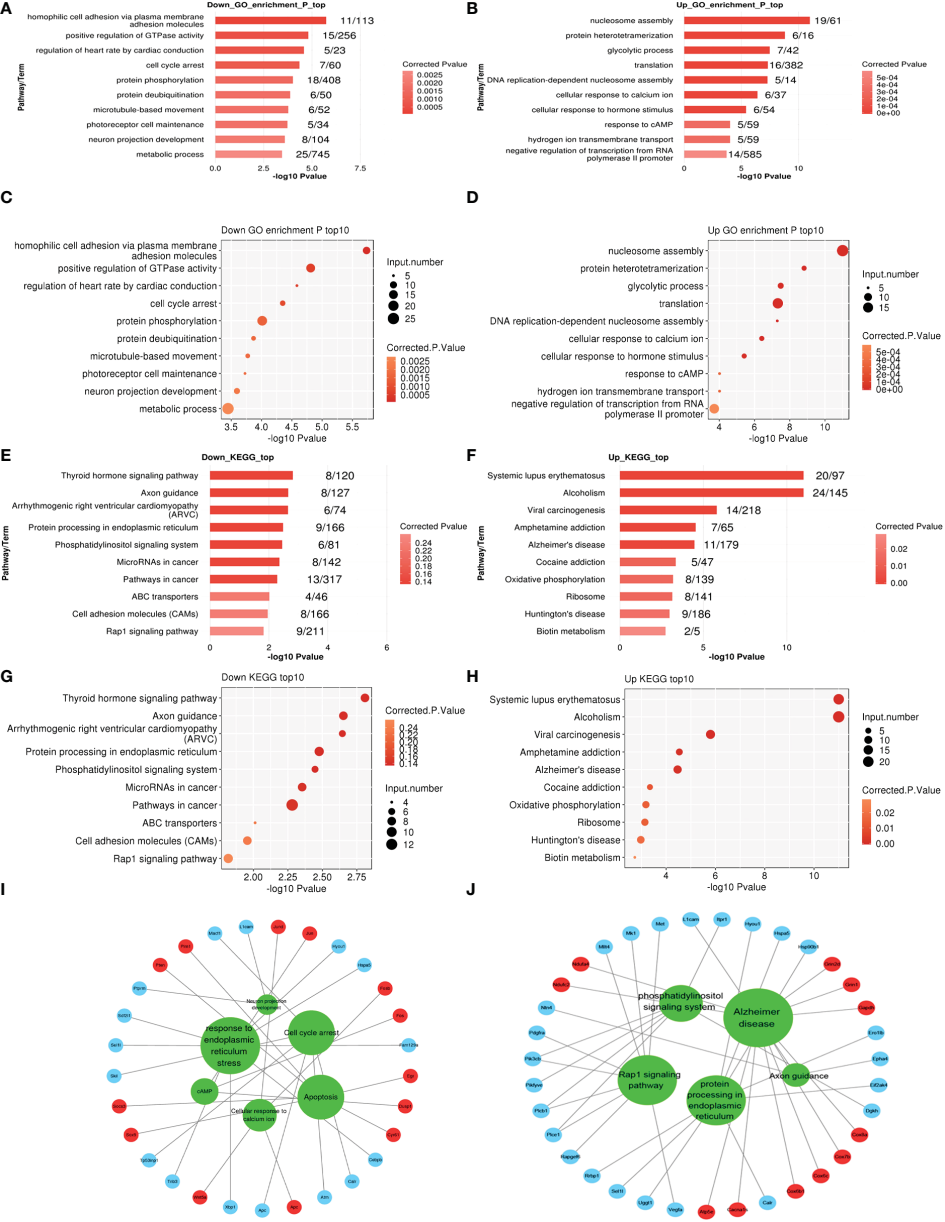
**(A–D)** The top ten enriched Gene Ontology (GO) biological process (BP), terms including down and upregulation (1 kPa vs. 50 kPa) in R28 cells. **(E–H)** The top ten enriched KEGG pathway terms associated with down and upregulation in R28 cells. **(I, J)** The interaction networks of all significantly enriched GO or KEGG and related genes in R28. The red circles are upregulated genes, and blue circles are downregulated genes linked to the GO or KEGG, indicated in the green circles.

The expression analysis was used to examine the changes that occurred in cells between 50 and 1.1 kPa. GO analysis showed that in the cell model, 75 GO terms were significantly downregulated, and 30 GO terms were significantly upregulated and associated with biological processes. The top 10 GO terms that were significantly upregulated and downregulated are listed in **Figures 2A–D**, respectively. KEGG pathway analysis showed that 191 pathways in the cell model were significantly downregulated, whereas 150 pathways were significantly upregulated. The top ten signaling pathways that were upregulated and downregulated are shown in **Figures 2E–H**, respectively. Signaling pathways related to axons were significantly downregulated in cell models, whereas signaling pathways related to various neurodegenerative diseases (such as Alzheimer’s and Huntington’s disease) were significantly upregulated. Additionally, genes related to these pathways were identified (**Figures 2I, J**).

### 3.3 Expression patterns of genes that respond to substrate stiffness cluster with functionally enriched pathways

**Figure 3:**
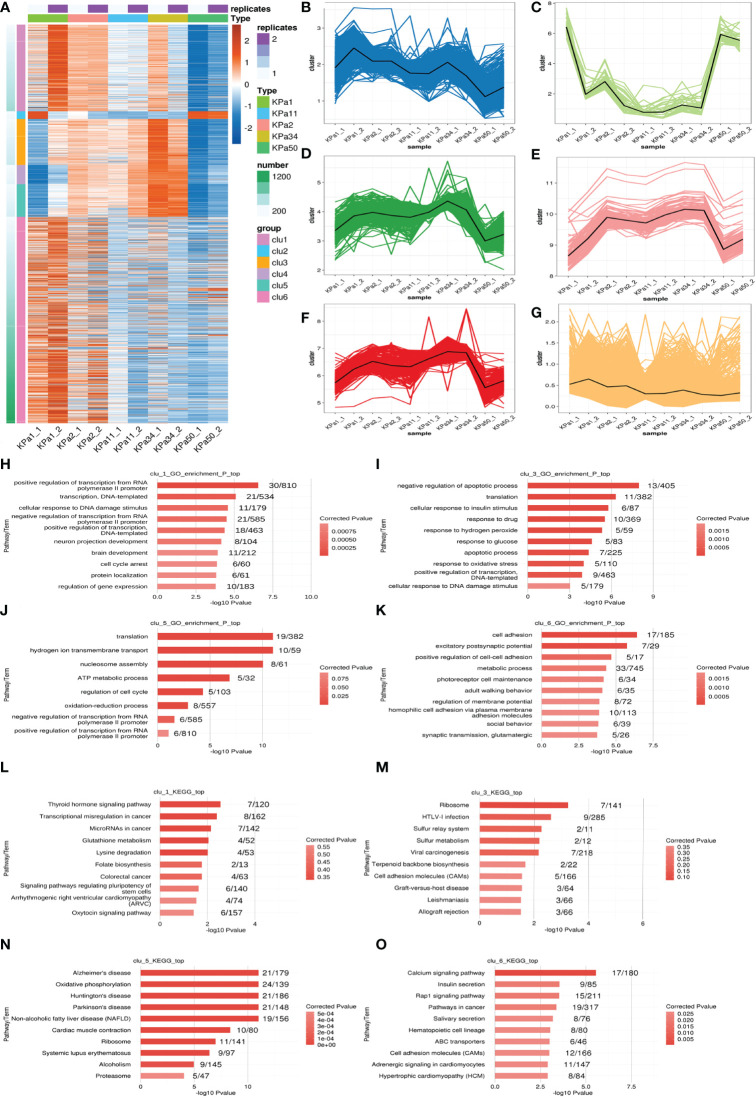
**(A)** k-means clustering of differentially expressed lncRNAs. **(B–G)** Genes are separated into 6 clusters, the black line represents the average expression value of all genes in each cluster. **(H–K)** Ten GO terms with significant differential expression identified from GO analysis of the biological processes represented in the different clusters. **(L–O)** The top ten KEGG pathways with significant expression differences in the identified clusters.

Gene expression cluster analysis can be used to identify genes with similar expression patterns and functions. Therefore, we performed k-means cluster analysis on the DEG of lncRNAs in the cell model to identify genes with similar expression patterns (**Figure 3A**). We identified six main expression patterns (**Figures 3B–G**), that represented the gene responses to increased substrate stiffness. The expression characteristics of clusters 1 and 6 were similar, which illustrated that the expression levels gradually decreased with increasing pressure. The expression characteristics of clusters 3 and 5 were very similar, indicating that the expression levels gradually increased with increasing pressure, and this expression level was observed again at 50 kPa. Functional cluster analysis was performed on different gene clusters to obtain their respective enrichment functions. Clusters 1 and 6 were mainly enriched in pathways related to neuro-projection development, synaptic transmission, and glutamatergic pathways, whereas clusters 3 and 5 were mainly enriched in pathways related to apoptosis, oxidative stress, and ATP metabolism (**Figures 3H–K**). Clusters 2 and 4 had fewer genes and enriched functions. In the KEGG analysis, we focused on the enrichment of pathways related to neurodegenerative diseases in cluster 5, which is consistent with a previous report that glaucoma may be a neurodegenerative disease of the retina (**Figures 3L–O**) (15).

### 3.4 Construction of a lncRNA-mRNA co-expression network

**Figure 4:**
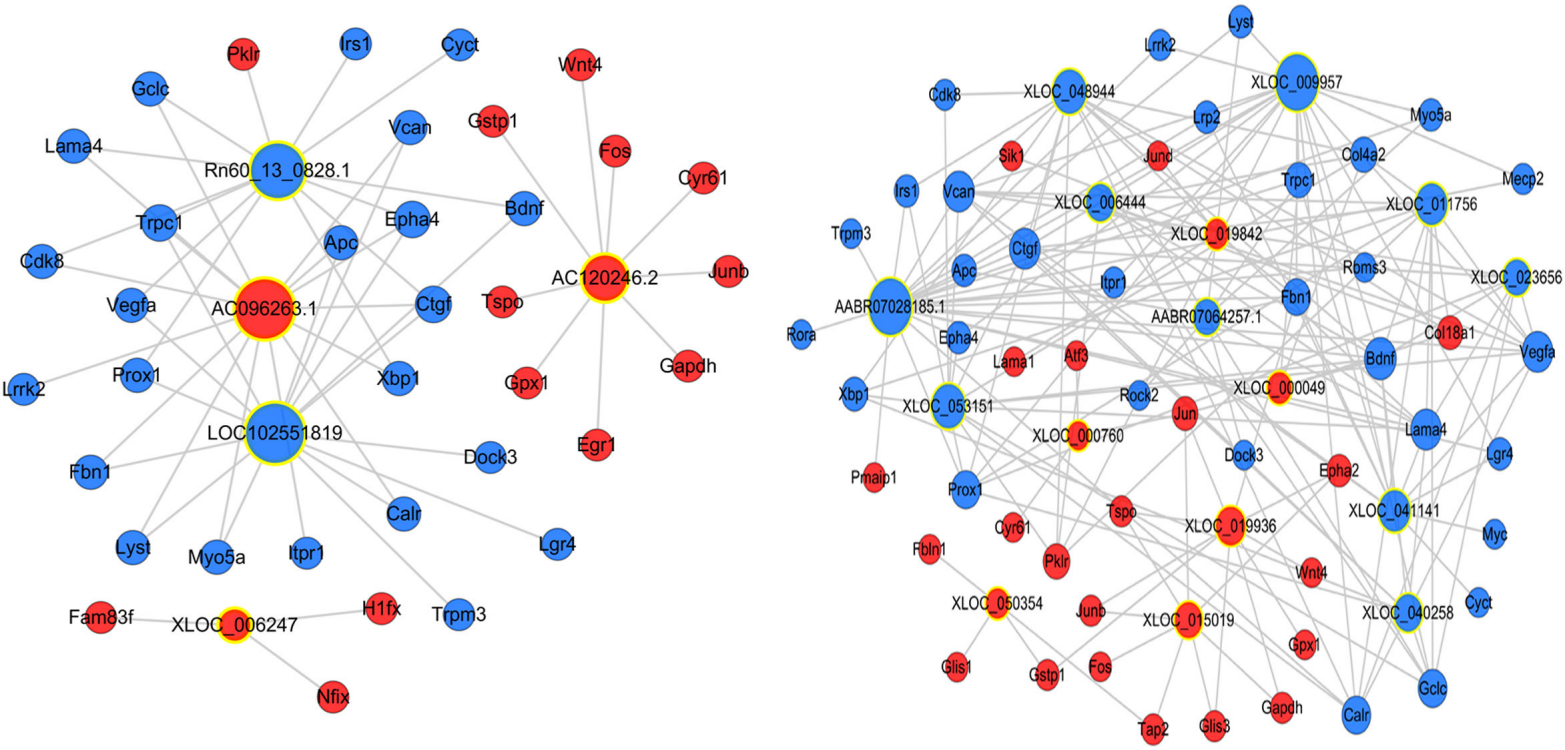
Construction of the lncRNA-mRNA co-expression network. A node with a yellow ring indicates lncRNA and a node without a yellow ring indicates mRNA. Upregulated lncRNAs and mRNAs are shown in red and downregulated lncRNAs and mRNAs are shown in blue.

The potential interaction between mRNA and lncRNAs was explored by establishing an lncRNA-mRNA co-expression network. We confirmed that 333 DElncRNAs were co-expressed with 1153 DEmRNAs (Pearson’s correlation coefficient analysis > 0.99 or < -0.99). Some lncRNAs were selected with obvious and consistent expression changes from expression clusters 1, 3, 5, and 6, and constructed a simple lncRNA-mRNA network for further research (**Figure 4**).

### 3.5 Construction of a competing endogenous RNA network

**Figure 5:**
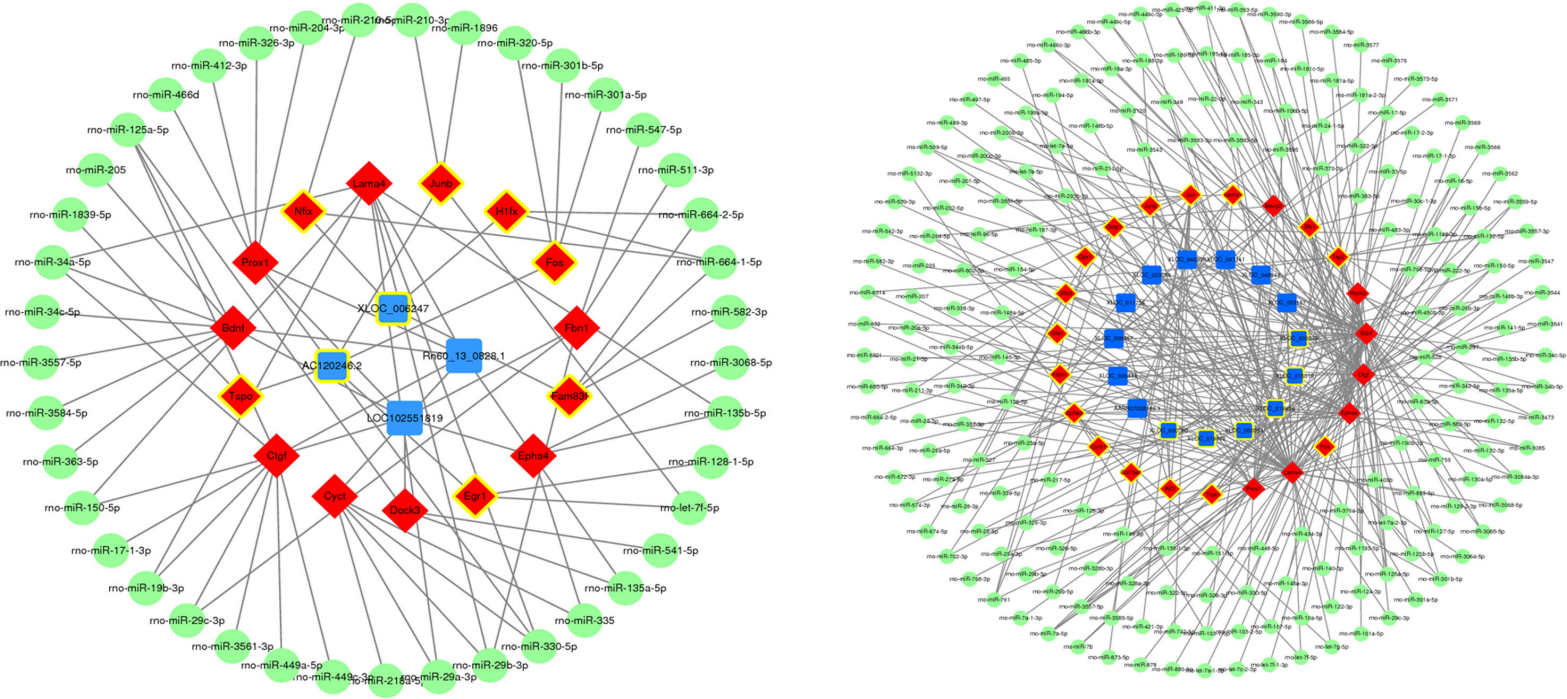
Construction of competitive endogenous RNA networks. Nodes with yellow circles indicate upregulated RNA, while nodes without yellow circles indicate downregulated RNA. lncRNA, mRNA, and miRNA are shown in blue, red, and green respectively.

A ceRNA network was constructed to reveal interactions between miRNAs, mRNAs, and lncRNAs. Among the four lncRNAs that we focused on, rho-miR-34a-5p, rho-miR-125a-5p, rho-miR-664-1-5p, rho-miR-330-5p, and rho-miR-29b-3p were enriched in this ceRNA network . These results indicate that these five miRNAs may play important roles in the regulation of glaucoma-related genes. Additionally, the ceRNA network diagram of the remaining lncRNAs showed a change in expression during glaucoma (**Figure 5**).

### 3.6 Candidate lncRNA screening and clinical sample verification

**Figure 6:**
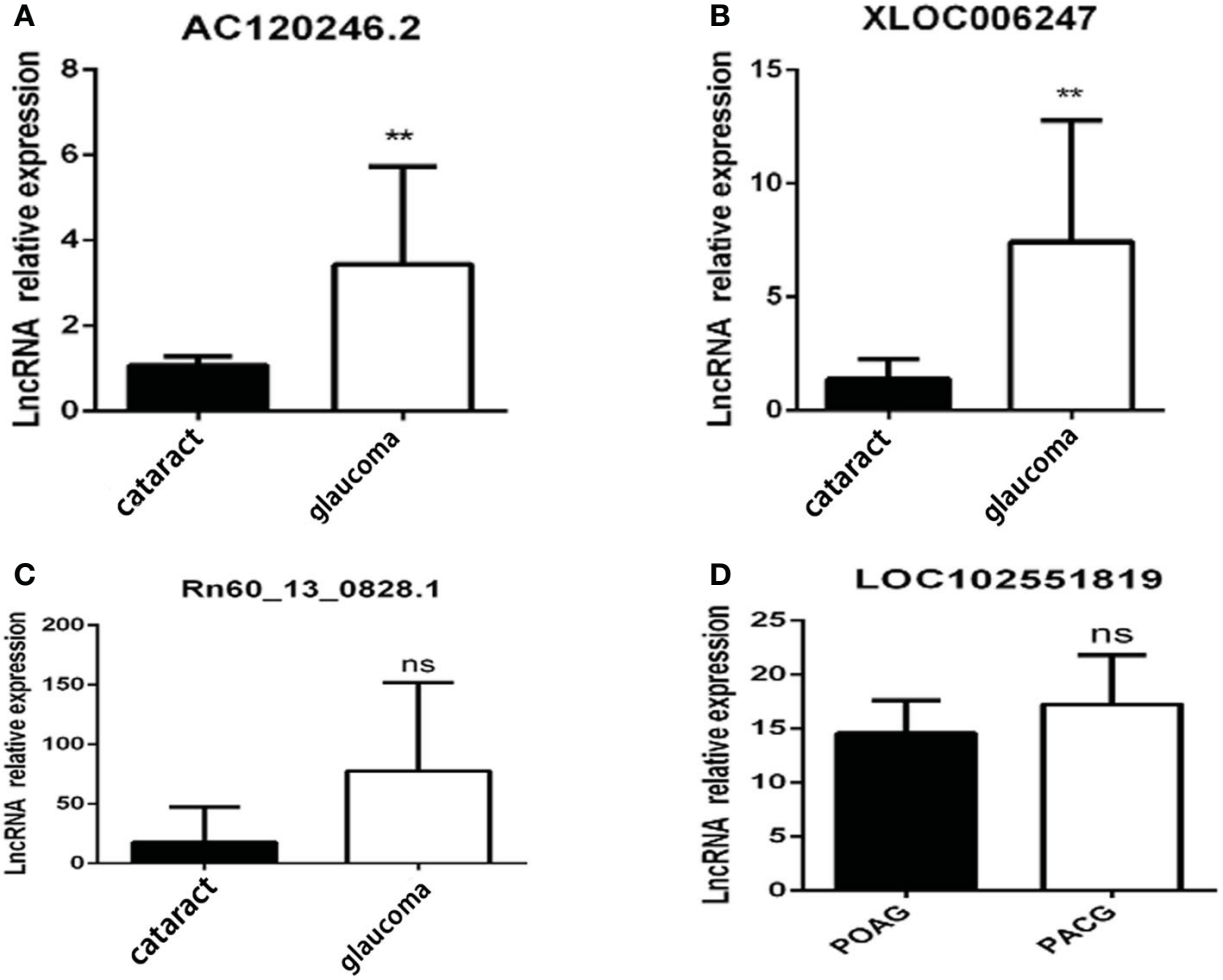
Candidate lncRNA expression in clinical patients. Candidate lncRNA expression in the aqueous humor **(A–C)** or iris **(D)** from patients with glaucoma(n=10) and patients with cataracts(n=5). **p < 0.01, ns p >0.05.

Clinically collected samples were used to verify the sequencing results. The lncRNA expression levels of AC120246.2 and XLOC_006247 in the AH of patients with glaucoma were significantly higher than those of patients without glaucoma (**Figures 6A, B**). LOC102551819 was observed to be specifically expressed in the iris. LOC102551819 expression was not detected in the AH and trabecular meshwork of patients with glaucoma. However, high expression was found in the iris tissue of patients with glaucoma, although no difference was observed between primary open-angle glaucoma and primary angle-closure glaucoma (**Figure 6D**). Rn60_13_0828.1 showed no significant differences in the test results from clinical samples (**Figure 6C**).

## 4 Discussion

The various biological effects and wide distribution of lncRNAs in the human body make them similar to other functional proteins. Currently, research on the specific mechanisms of lncRNAs in the pathogenesis of glaucoma is still in its infancy. The discovery of more meaningful lncRNAs in glaucoma is of great value for subsequent in-depth research. We performed RNA-seq on different samples from cell and mouse models and performed GO, KEGG, and k-means cluster analyses on the obtained results. We screened four of these lncRNAs with the closest expression, AC120246.2, XLOC_006247, Rn60_13_0828.1, and LOC102551819, and one previously reported lncRNA, AC120246.2, to perform RT-qPCR verification on clinical samples from patients with glaucoma and cataracts. Differential expression of these lncRNAs was observed between patients with glaucoma and patients without glaucoma, as well as between patients with POAG and patients with PACG, which may have potential applications in future clinical diagnosis and treatment. Previous reports have identified many DElncRNAs and DEmRNAs using RNA-seq based on the construction of a stress model in human trabecular meshwork cells (8, 11). The pathological progression of glaucoma is related to damage of Trabecular Meshwork (TM) cells regardless of the type of glaucoma; therefore, retinal cell damage and visual field loss will eventually occur. Our results are mainly based on data obtained from sequencing of retinal precursor cells. qRT-PCR verification of the clinical trabecular meshwork, iris, AH, and other tissue samples was also performed.

This study showed that there were many DElncRNAs and DEmRNAs in pressurized R28 cells. Moreover, GO and KEGG analyses identified cellular events, biological processes, and signaling pathways related to glaucoma development. GO analysis revealed several obvious changes in biological processes related to the pathology of glaucoma, including cellular response to calcium ions, neuron projection development, cAMP, response to endoplasmic reticulum stress, cell cycle arrest, and apoptosis. The typical related pathways or BP differences obtained by KEGG analysis in cells were the phosphatidylinositol signaling system, Alzheimer’s disease, axon guidance, protein processing in the endoplasmic reticulum, and Rap1 signaling. The ECM-receptor interaction and PI3K-AKT, NF-κB, MAPK, and TNF signaling pathways were identified in mice. These associated pathways have been reported in many previous glaucomatous studies, most of which are related to RGC apoptosis (16, 17), trabecular meshwork dysfunction, and extracellular matrix proliferation (18). The co-expression results of the 21 lncRNAs and their corresponding mRNAs as an interaction network are displayed in the current study. Many of the genes associated with this network, such as *Jun*, *Jund*, *Apc*, *Xbp1*, *Fos*, and *Calr*, were consistent with the BP-related mRNAs. Among these, *Jun*, *Jund*, *Xbp1*, *Fos*, and *Calr* are associated with endoplasmic reticulum stress, astrocyte activation, and RGC apoptosis (19–22). *Apc* is related to human tenon fibroblast proliferation and can induce postoperative scarring of the glaucoma filter passage (23). It has been suggested that lncRNAs participate in biological processes related to glaucoma through direct or indirect interactions with these mRNAs.

lncRNAs function through multiple mechanisms, including the ceRNA hypothesis. This hypothesis states that lncRNAs, mRNA transcripts, and false gene transcripts can affect each other by competitively combining with MREs to influence post-transcriptional regulation (24). The ceRNA network links the functions of protein-coding mRNAs with non-coding RNAs such as miRNA, lncRNA, pseudogene RNA, and circular RNA. According to the ceRNA hypothesis, lncRNAs may act as miRNA “sponges” and compete with MREs, regulating miRNA-mediated biological processes (25, 26). We predicted miRNAs that interact with differentially expressed lncRNAs and mRNA in the course of glaucoma disease progression using ceRNA network constructions (**Figure 5**). The miRNAs rho-miR-34a-5p, rho-miR-125a-5p, rho-miR-664-1-5p, rho-miR-330-5p, and rho-miR-29b-3p were enriched in the ceRNA network, suggesting that they interacted with the largest number of DEmRNAs and DElncRNAs. Among them, rho-miR-34a-5p is a common miRNA with abnormal regulation in central nervous system diseases (27). Furthermore, rho-miR-125a-5p is related to ischemic stroke and neuronal differentiation (28, 29), and rho-miR-29b-3p targets genes such as *FOXO3a* and *TRAF5* in a cardiac ischemia-reperfusion model to protect cardiomyocytes from endotoxin-induced apoptosis and inflammation (30–32). Finally, rho-miR-330-5p and rho-miR-664-1-5p can also inhibit myocardial ischemia-reperfusion injury through targeted regulation (33, 34). The mechanism of ischemia reperfusion has also been shown to play a role in glaucoma pathogenesis (35). Recently, the hypothesis that glaucoma is a neurodegenerative disease has also gained scientific acceptance (36). Therefore, these miRNAs may play an important role in glaucoma pathogenesis, although the specific mechanism requires further investigation.

Aqueous humor, trabecular meshwork, and iris tissues were collected from patients with glaucoma and other types of patients and verified the lncRNA expression profiles. The expression of AC120246.2 and XLOC_ 006247 in the aqueous humor of patients with glaucoma increased significantly (**Figure 6**). The LOC102551819 lncRNA may be specifically expressed in the iris, as we did not detect its expression in the aqueous humor and trabecular meshwork of patients with glaucoma. Rn60_13_0828.1 showed no significant differences in the test results from clinical samples. The difference in expression of Rn60_13_0828.1 may be related to species differences or because the sample number of patients was insufficient; therefore, further investigation is needed to confirm these results.

In this study, we initially identified a few lncRNAs that may play a role in the pathogenesis of glaucoma, but did not delve into the mechanisms by which these lncRNAs are involved in the pathogenesis of glaucoma, which is an area that needs to be further explored in the future. lncRNAs are involved in the pathogenesis of glaucoma in three main ways, in addition to regulating the function of trabecular meshwork cells, participating in ECM and scar formation after glaucoma filtration surgery, and directly regulating RGC damage (37).It has been demonstrated that lncRNA may serve as a potential biomarker for primary open-angle glaucoma (38–40), and studies on the mechanism of lncRNA have mainly focused on the ceRNA mechanism. Several studies have demonstrated that lncRNA can regulate the loss of retinal ganglion cells (41–44), apoptosis of human trabecular meshwork cells and extracellular matrix deposition through the ceRNA mechanism (45–48). In the mechanistic studies of ceRNA or RBP, the current situation is that one lncRNA can target multiple molecules, and multiple lncRNAs can act on the same target. Therefore, when studying the disease development process at the molecular level, it should not be isolated to only one specific lncRNA, and targeting only this lncRNA may not achieve the desired therapeutic effect, and it may be necessary to use bioinformatics to network it and find the core lncRNA. However, lncRNAs act in a variety of ways. In addition to continuing to explore the role of lncRNAs in glaucoma through the ceRNA mechanism, the specific ways in which lncRNAs regulate glaucoma occurrence and development through other modalities should be more extensively explored in the future. Some studies have reported that some ncRNA can encode functional small proteins that are commonly referred to as small peptides (49, 50), highlighting the possibility that additional transcripts currently annotated as ncRNA encode proteins with important biological activity. This area is still unknown in glaucoma and also deserves to be studied and explored.

In conclusion, the expression profiles of lncRNA-mRNA associated with the pathogenesis of glaucoma was evaluated using RNA-seq and the underlying regulatory mechanism determined through bioinformatics analyses. We aimed to reveal the role of lncRNAs in glaucoma pathogenesis and our results may provide potential targets for the diagnosis and treatment of glaucoma. However, our study also has some limitations, such as the lack of functional studies on these DElncRNAs. Therefore, further research is needed to explore the role of these DElncRNAs in the pathogenesis of glaucoma.

## Data availability statement

The data presented in the study are deposited in the Sequence Read Archive of NCBI repository, accession numberPRJNA919494.

## Ethics statement

The studies involving human participants were reviewed and approved by Xiangya Hospital of Central South University. The patients/participants provided their written informed consent to participate in this study. The animal study was reviewed and approved by Xiangya Hospital of Central South University.

## Author contributions 

Conceptualization: DJ; Methodology: MY and CF; Formal analysis and investigation: RR, ZZ, HL, and QY; Writing- Original draft preparation, RR, MY, and ZZ; Writing- Reviewing and Editing: DJ. All authors contributed to the article and approved the submitted version.
