# Supplementary material for: Integrated analysis of long non-coding RNAs and mRNAs associated with glaucoma in vitro
Source: Front Endocrinol (Lausanne). 2023 Feb 27;14:1087442. doi: 10.3389/fendo.2023.1087442 (PMC10008935; doi:10.3389/fendo.2023.1087442)
Supplement: Supplementary file 1 [file Table_1.docx]

| Number | Age | Sex | Diagnosis |
| --- | --- | --- | --- |
| 1 | 68 | Male | Catatract |
| 2 | 72 | Female |  |
| 3 | 71 | Male |  |
| 4 | 64 | Male |  |
| 5 | 65 | Female |  |
| 1 | 71 | Male | Glaucoma |
| 2 | 72 | Female |  |
| 3 | 65 | Male |  |
| 4 | 68 | Female |  |
| 5 | 73 | Female |  |
| 6 | 62 | Male |  |
| 7 | 65 | Female |  |
| 8 | 66 | Male |  |
| 9 | 77 | Male |  |
| 10 | 73 | Male |  |

Table S1 Clinical characteristic of patients with glaucoma and cataract
