# Supplementary material for: Integrated analysis of long non-coding RNAs and mRNAs associated with glaucoma in vitro
Source: Front Endocrinol (Lausanne). 2023 Feb 27;14:1087442. doi: 10.3389/fendo.2023.1087442 (PMC10008935; doi:10.3389/fendo.2023.1087442)
Supplement: Supplementary file 2 [file Table_2.docx]

Table S2 Primer sequences used in q-PCR experiments

| **Gene name** | **Prime sequence（5**^，^ **3**^，^**）** |
| --- | --- |
| **GAPDH** | F: GAGAAGGCTGGGGCTCATTT |
|  | R: AGTGATGGCATGGACTGTGG |
| **AC120246.2** | Povided by Ribobio |
| **XLOC_006247** | Povided by Ribobio |
| **LOC102551819** | Povided by Ribobio |
| **Rn60_13_0828.1** | Povided by Ribobio |
